# Supplementary material for: The lipid sensor GPR120 promotes brown fat activation and FGF21 release from adipocytes
Source: Nat Commun. 2016 Nov 17;7:13479. doi: 10.1038/ncomms13479 (PMC5118546; doi:10.1038/ncomms13479)
Supplement: Supplementary Information — Supplementary Figures 1-13, Supplementary Tables 1-3. [file ncomms13479-s1.pdf]

a

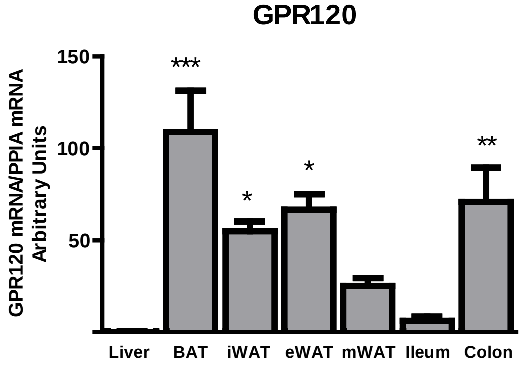

b

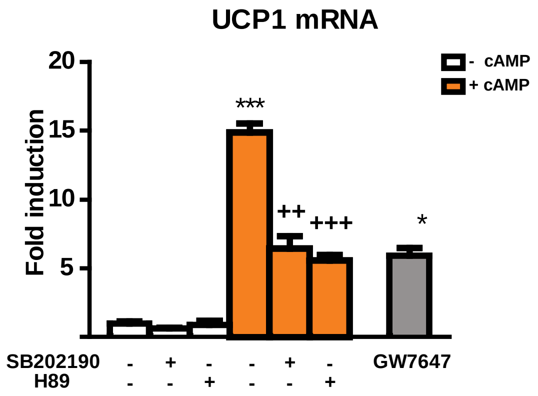

Supplementary Figure 1. GPR120 expression and effects of p38 MAPK and PKA inhibitors and PPARα agonist on UCP1 mRNA levels. (a) *GPR120* mRNA expression in mouse tissues using cyclophilin mRNA as housekeeping reference gene. Bars are means + s.e.m. (*P <0.05, **P <0.01, ***P <0.001 compared with ileum; two-tailed unpaired student t-test). **(b)** Effects of 10 μM SB202190 (a p38 MAPK inhibitor), or 20 μM H89 (a PKA inhibitor) on the up-regulation of *UCP1* mRNA in response to 1mM dibutyryl-cAMP, and effects of 1 μM GW7647 (PPARα agonist) in brown adipocytes differentiated in primary culture (n = 4). Bars are means + s.e.m. (*P <0.05, ***P <0.001 compared with controls; ++P<0.01, +++ P<0.001 for the effects of SB202190 or H89; ANOVA with Tukey's post hoc test).

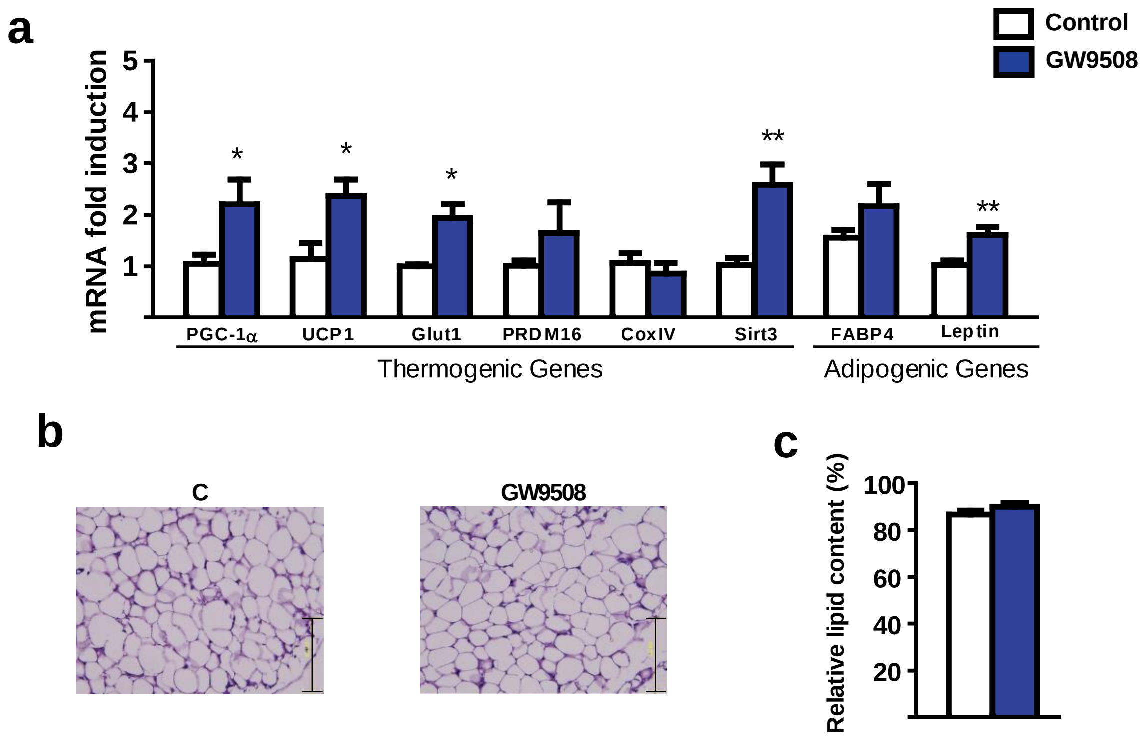

Supplementary Figure 2. Effects of GW9508 on thermogenic genes in epididymal WAT. Adult mice were fed for 7 days a control diet (white bars) or a diet supplemented with GW9508 (blue bars) (n = 6). **(a)** Relative expression levels of thermogenic and adipogenic genes in eWAT, **(b)** representative optical microscopy images from H&E-stained eWAT (scale, 125µm), **(c)** relative lipid content. Bars are means + s.e.m. (*P<0.05 and **P<0.01relative to untreated control mice; two-tailed unpaired student t-test).

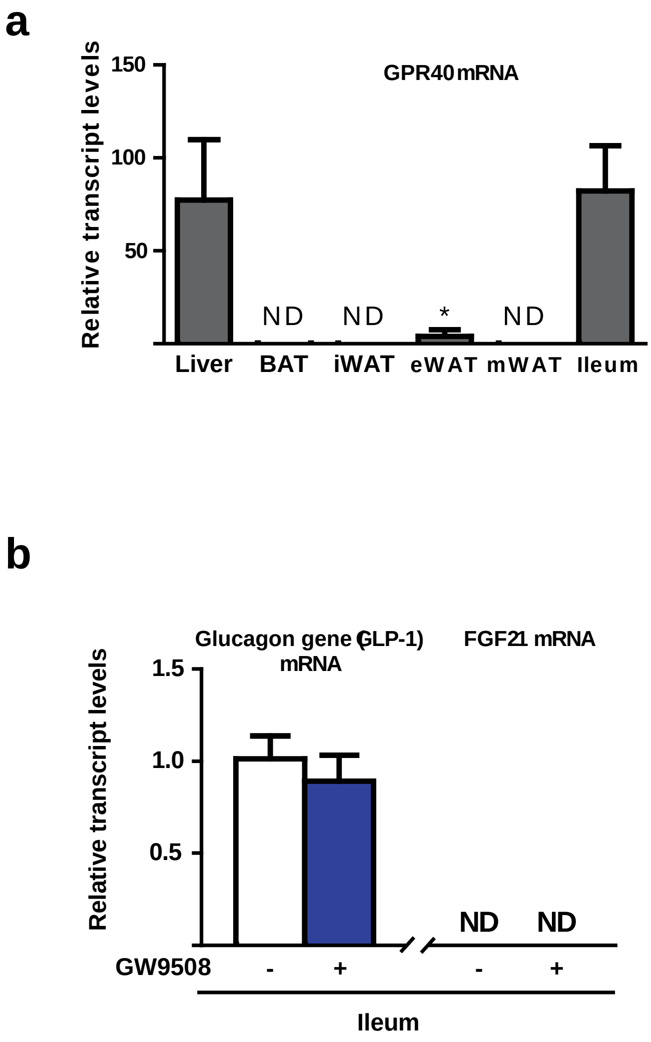

Supplementary Figure 3. GPR40 mRNA expression levels in adipose depots and GW9508 effect on intestinal cells. (a) *GPR40* mRNA expression levels in mouse tissues (n = 4). **(b)** Relative expression levels of *GLP-1* and *FGF21* in Ileum from adult mice fed for 7 days a control diet (white bars) or a diet supplemented with GW9508 (blue bars). Bars are means + s.e.m. (*P<0.05 relative to liver GPR40 mRNA expression; two-tailed unpaired student t-test).

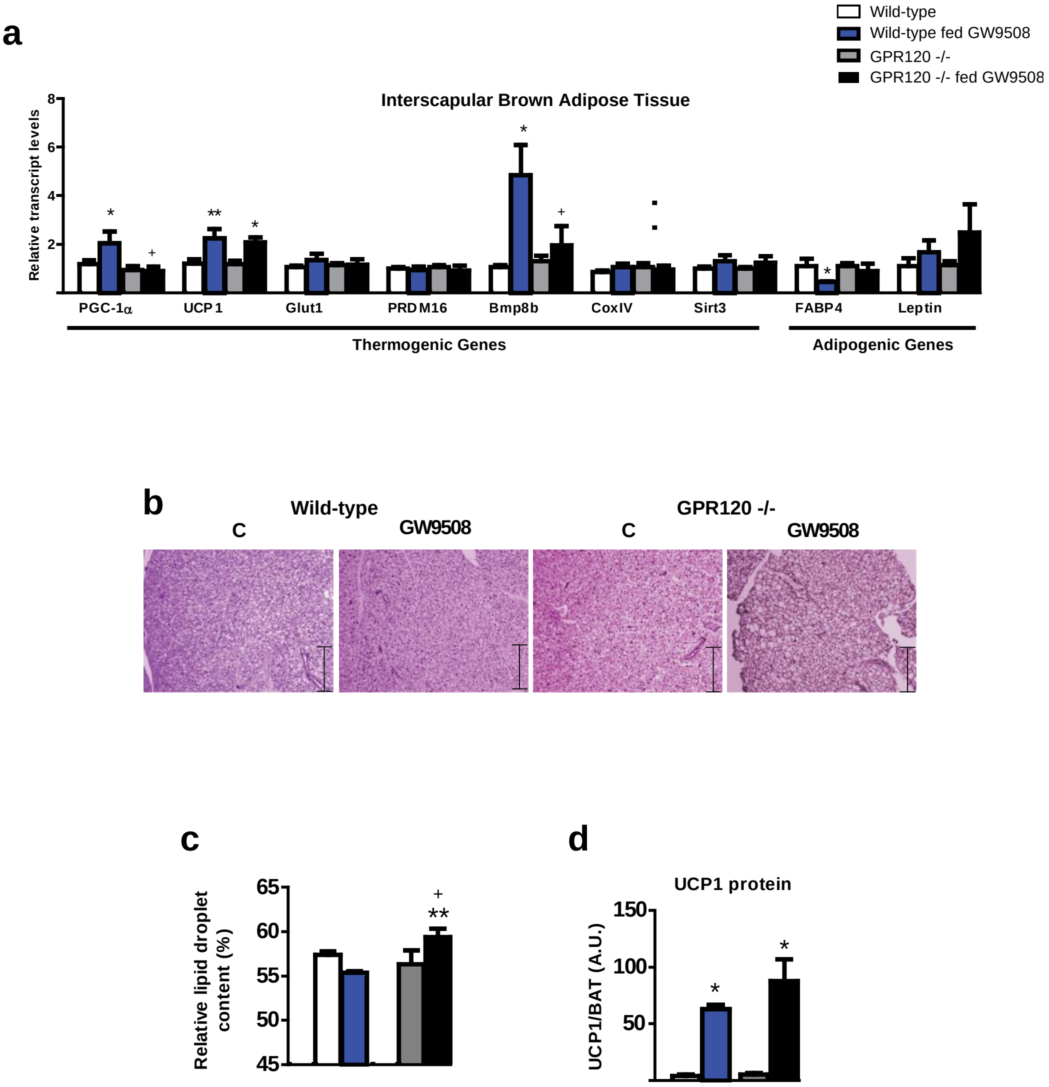

Supplementary Figure 4. Effects of GPR120 gene invalidation on the response to GW9508 treatment in iBAT. *Wild-type* and *GPR120-/-* mice (n = 5) were fed for 7 days a control diet (white and grey bars, respectively) or a diet supplemented with GW9508 (blue and black bars, respectively). **(a)** Relative expression levels of thermogenic and adipogenic genes in iBAT. **(b)** Representative optical microscopy from H&E-stained iBAT (scale, 200µm), **(c)** relative lipid content, **(d)** UCP1 protein levels. Bars are means + s.e.m. (*P<0.05, **P<0.01, and ***P<0.001 relative to untreated mice of each genotype; +P<0.05, ++P<0.01 relative to the effects of the genotype in mice under the same treatment; ANOVA with Tukey's post hoc test).

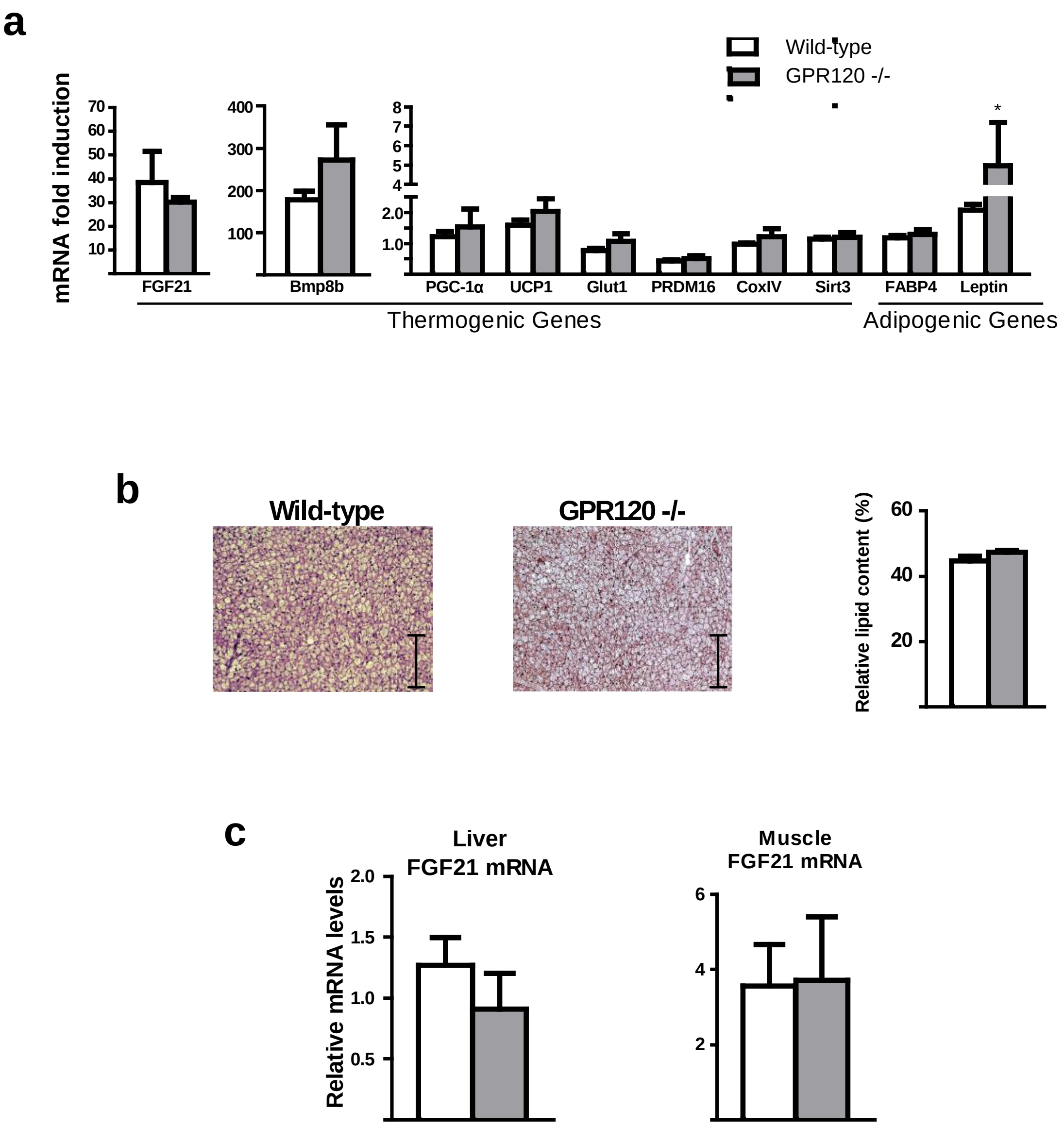

Supplementary Figure 5. Effects of GPR120 invalidation on iBAT from mice exposed to cold. *Wild-type* (white bars) and *GPR120-/-* (grey bars) mice were exposed to cold (4ºC) for 7 days (n=5). **(a)** Relative expression levels of thermogenic and adipogenic genes in iBAT. **(b)** Representative images of H&E-stained iBAT (scale, 125µm) and the relative lipid droplet content. **(c)** *FGF21* mRNA expression levels in liver and skeletal muscle. Bars are means + s.e.m. (*P<0.05, relative to wild-type control mice; two-tailed unpaired student t-test).

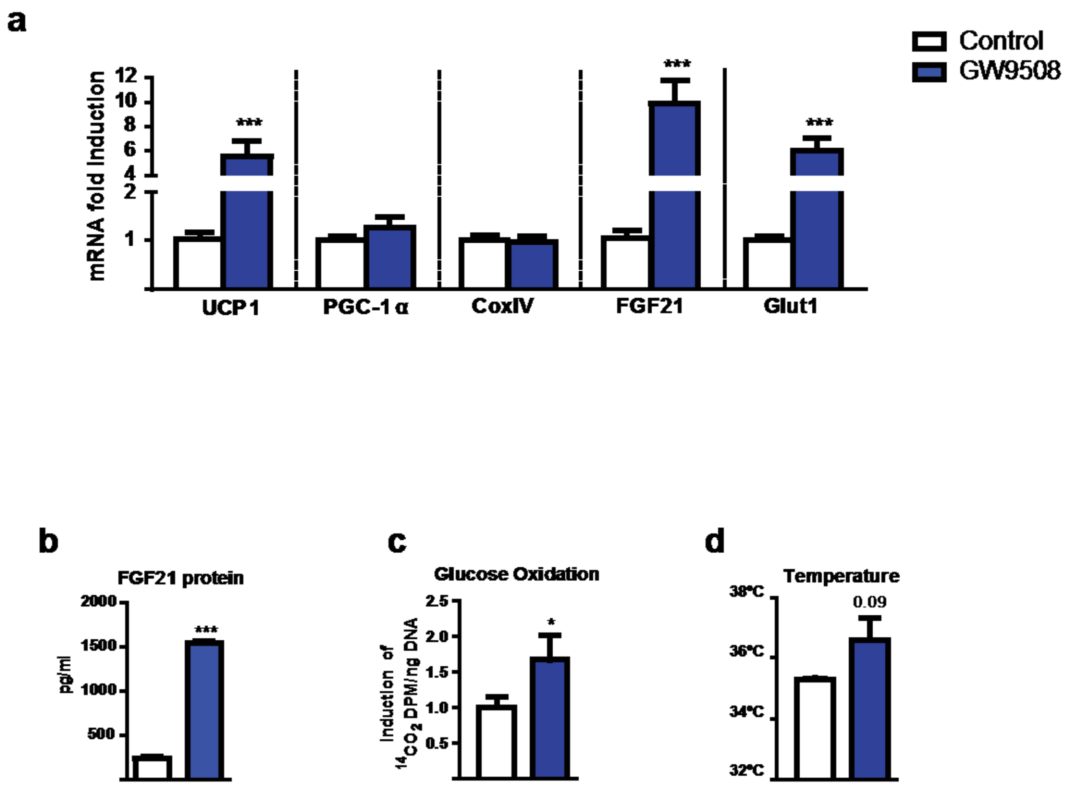

Supplementary Figure 6. GPR120 activation induces thermogenesis-related genes expression, glucose oxidation and FGF21 protein release in brown adipocytes. iBAT precursors were differentiated and treated for 24h with 100µM GW9508 (n = 5) **(a)** Relative expression levels of *UCP1*, *PGC-1α*, *CoxIV*, *FGF21* and *Glut1*. **(b)** FGF21 protein in supernatant (24h accumulation). **(c)** Glucose oxidation rate. **(d)** Cell culture temperature. Bars are means + s.e.m. (*P<0.05, **P<0.01, and ***P<0.001 relative to untreated control cells; two-tailed unpaired student t-test).

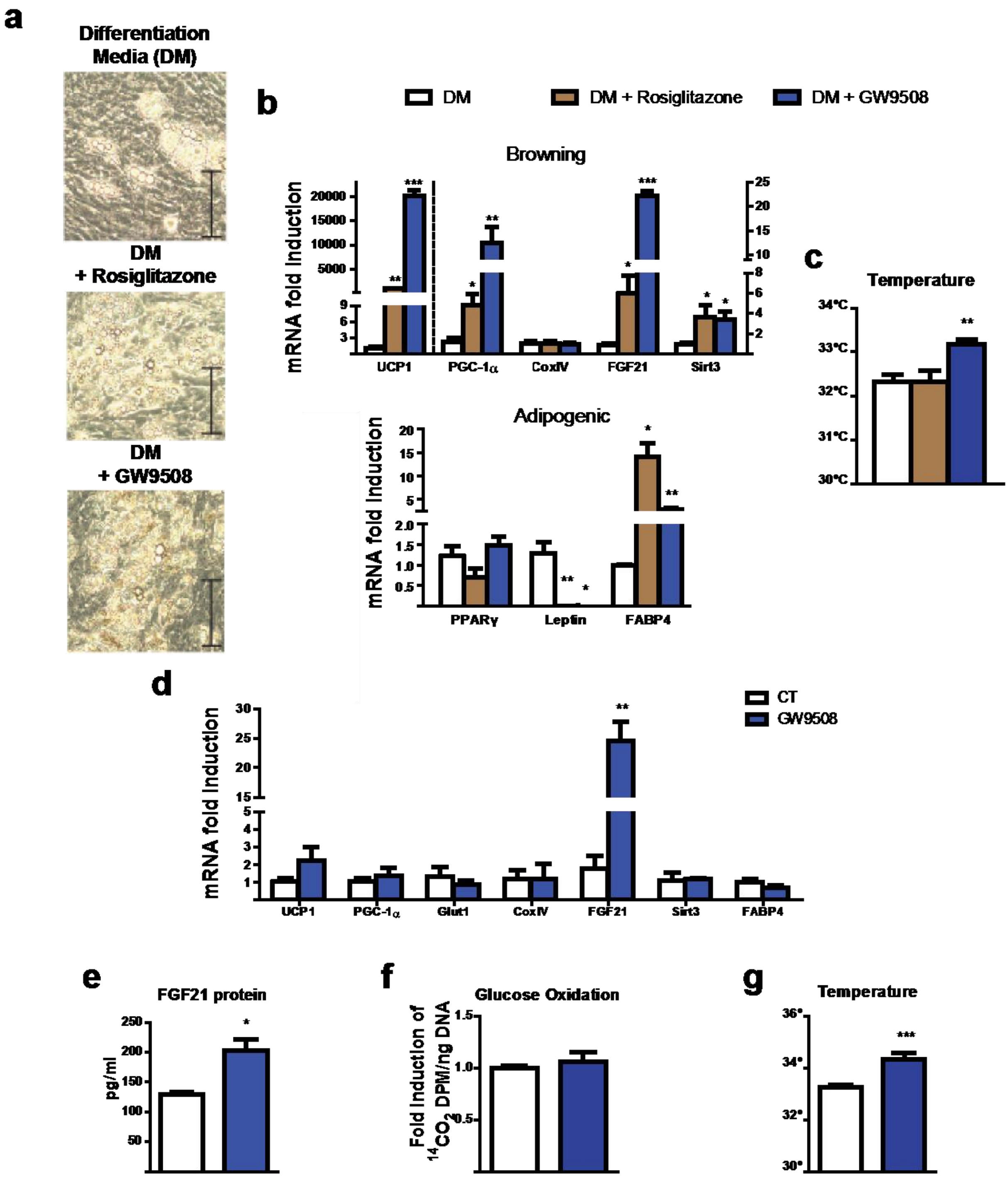

Supplementary Figure 7. GPR120 activation promotes eWAT derived beige adipocyte differentiation and increases FGF21 expression and release. For a-c, eWAT precursors from wild-type mice were differentiated (n=3-5) in the presence of the differentiation media (DM) (n=3), supplemented with rosiglitazone to drive beige differentiation (DM + Rosiglitazone)(n=5), or treated with GW9508 instead of rosiglitazone (DM + GW9508) (n=5) (see Methods section). **(a)** Representative optical microscopy images at the end of differentiation (day 7) (scale bar, 200µm). **(b)** Relative mRNA expression levels of browning-related and general adipogenic genes. **(c)** Cell culture temperature. For d-g, eWAT precursors from *wild-type* mice were differentiated and treated during 24h with GW9508 (100µM) (n=5). **(d)** mRNA expression levels of *UCP1*, *PGC-1α*, *Glut1*, *COXIV*, *FGF21*, *Sirt3*, and *FABP4*. **(e)** FGF21 protein levels in culture media (24h accumulation). **(f)** Glucose oxidation rate. **(g)** Cell culture temperature. Bars are means + s.e.m. (*P<0.05, **p<0.01, and ***p<0.001 versus controls; two-tailed unpaired student t-test).

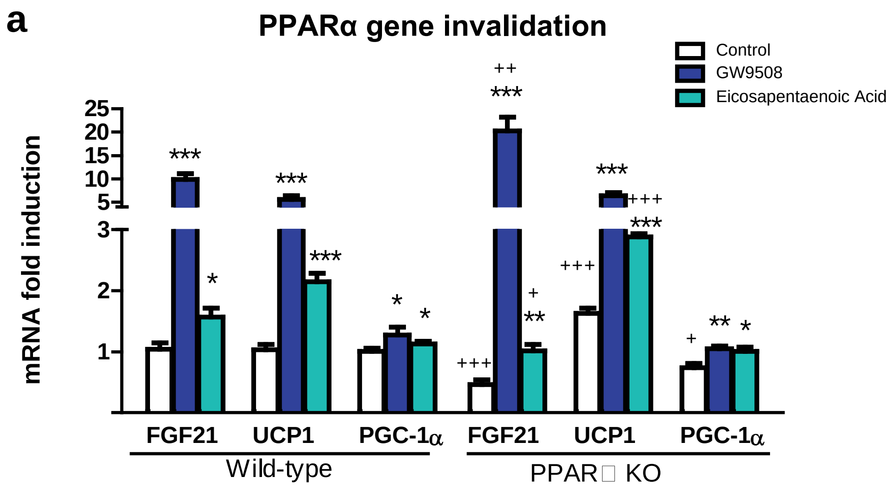

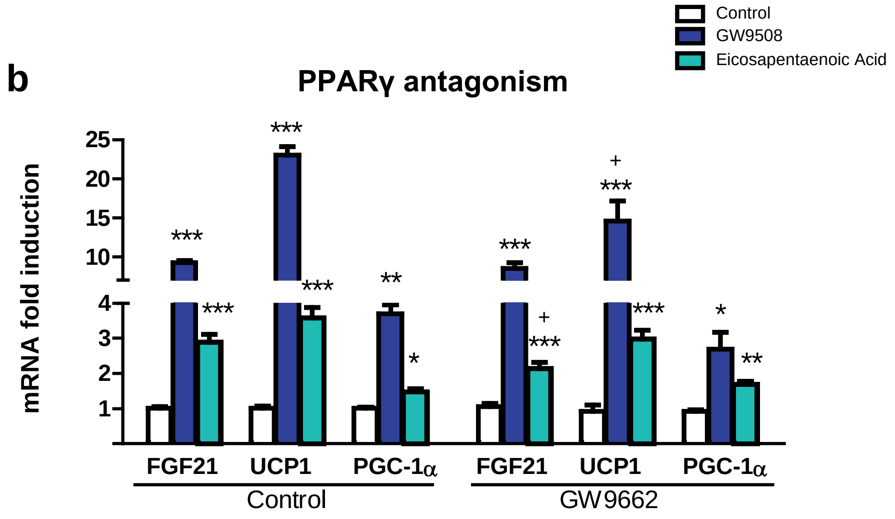

Supplementary Figure 8. Involvement of the PPARα and PPARγ pathways in the FGF21 induction elicited by activation of GPR120. (a) iBAT precursors from *wild-type* and *PPARα–null* mice were differentiated and treated during 24h with GW9508 (100µM) or EPA (100µM). Relative mRNA expression for *FGF21*, *UCP1* and *PGC-1α* is shown (n = 3). **(b)** iBAT precursors from wild-type mice were differentiated and treated with GW9508 or EPA and the PPARγ antagonist (GW9662). Relative mRNA expression for *FGF21*, *UCP1* and *PGC-1α* is shown (n = 3). Bars are means + s.e.m. (*P<0.05, **p<0.01, and ***p<0.001 versus controls; +P<0.05, ++p<0.01, and +++p<0.001 PPARα-null cells or GW9662 treated cells; ANOVA with Tukey's post hoc test).

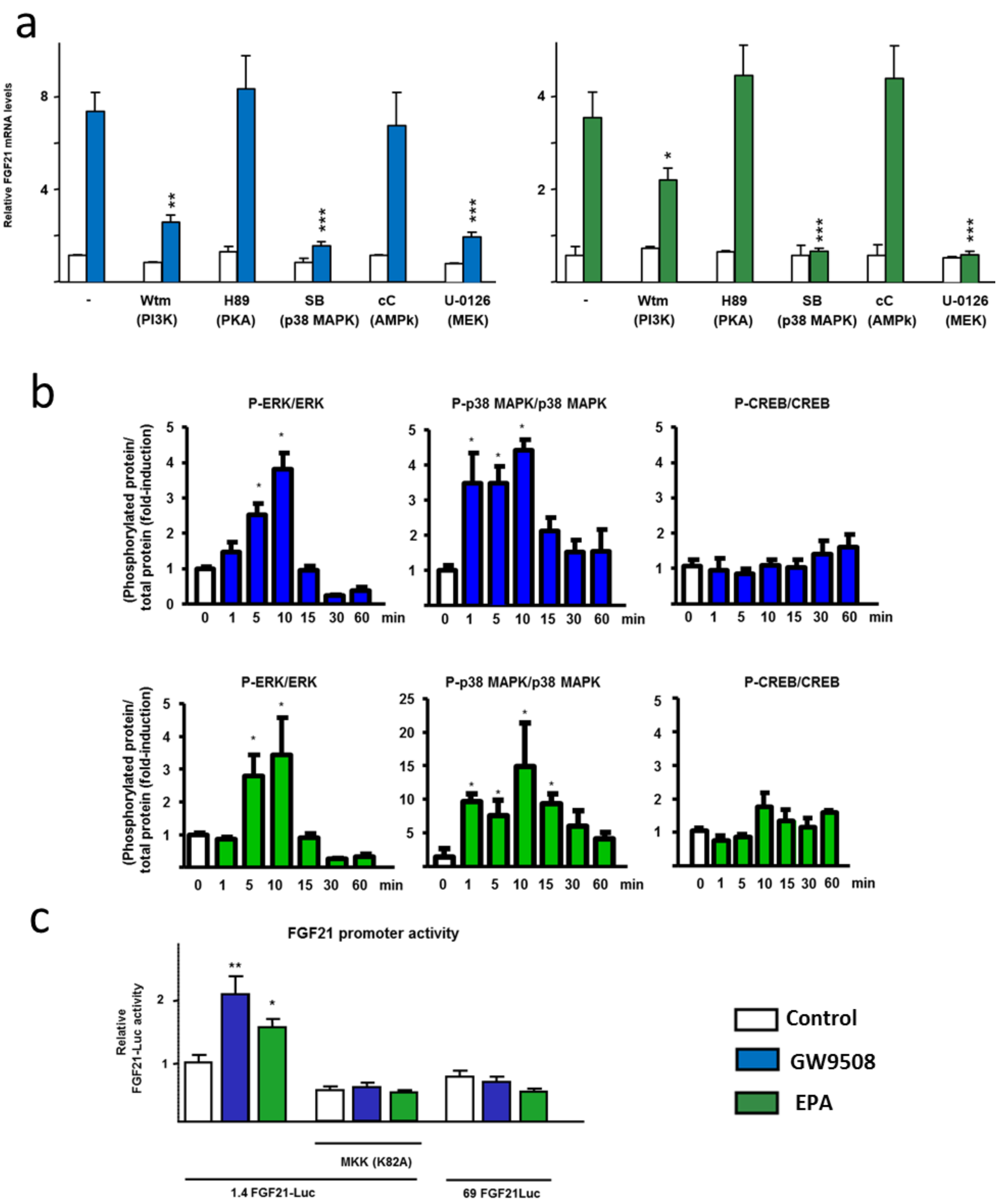

Supplementary Figure 9. Effects of GW9508 or EPA on FGF21 mRNA expression in the presence of kinases inhibitors, phosphorylation of regulatory kinases, and FGF21 gene promoter activity. Brown adipocytes in culture were used. **(a)** *FGF21* mRNA expression after 24h treatment with GW9508 (100μM) or EPA (100μM) in the presence of 2 μM wortmannin (PI3K inhibitor), 10 μM compound C (AMP kinase inhibitor),10 μM SB202190 (p38 MAPK inhibitor), 20 μM H89 (PKA inhibitor), 10 μM U-0128 (ERK inhibitor) (n = 4). **(b)** Relative levels of phosphorylated versus total kinases in response to GW9508 or EPA at the indicated times of treatment (n = 3). **(c)** *Wild-type* (1.4 FGF21-Luc) and deleted (69-FGF21-Luc) FGF21 gene promoter activity in transfected brown adipocytes in response to GW9508 or EPA. Effects of co-transfection with the dominant negative form of MKK6 (K82A) (n = 4). Bars are means + s.e.m. (*P<0.05, versus controls in the absence of inhibitors in (a), and versus non-treated cells in (b) and (c); for a, Student's t test was used and for b and c, ANOVA and Tukey post-hoc tests.

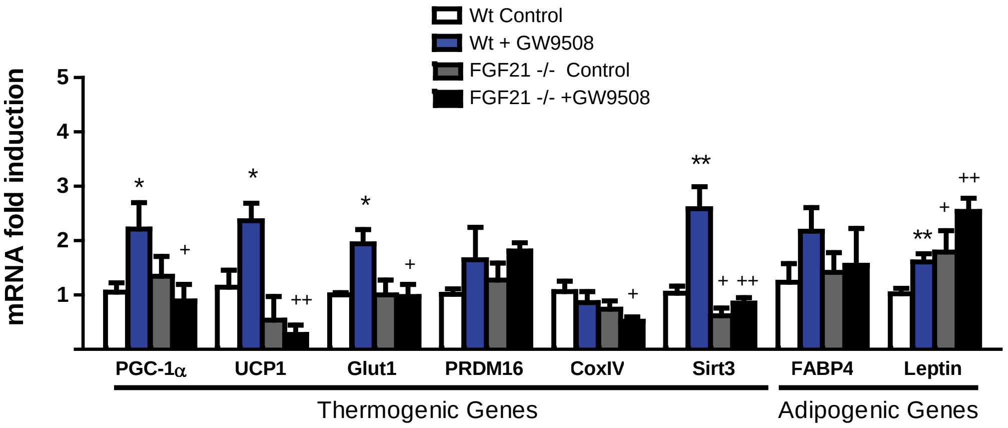

Supplementary Figure 10. Impaired effects of GW9508 on FGF21-null beige adipocytes derived from eWAT. eWAT precursors from *wild-type* and *FGF21-null* mice were differentiated and treated with GW9508 (24h) (n = 5), mRNA expression levels of thermogenic and adipogenic genes are shown. Bars are means + s.e.m. (*P<0.05, **P<0.01, for the effects of GW9508; and +P<0.05, ++P<0.01, for comparisons between *wild-type* and *FGF21-null* cells; ANOVA with Tukey's post hoc test).

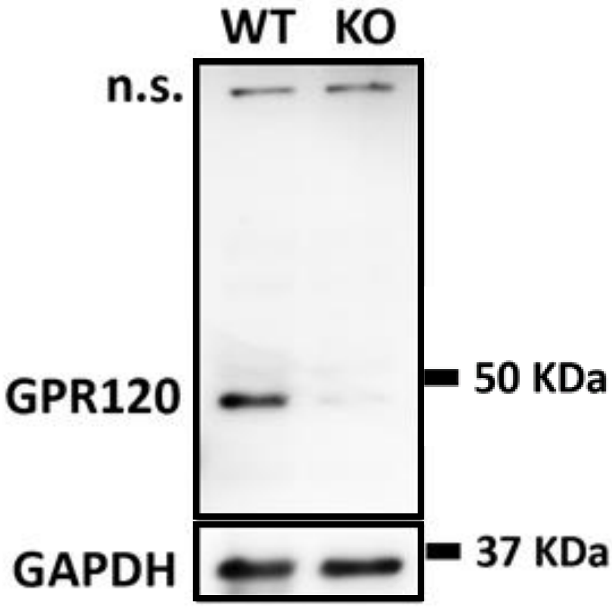

Supplementary Figure 11. Validation of specificity of GPR120 detection by immunoblot using BAT from GPR120-null mice. The Figure shows a representative western blot of 20 µg of protein extracts from mouse iBAT from *wild-type* (WT) and *GPR120-null* (KO) mice probed with the sc-99105 rabbit anti-GPR120 antibody (1:150) (Santa Cruz, USA). N.s., non-pecific. GAPDH, probing of the membrane with the leading control GAPDH (1:3000) to ensure equal loading of lanes.

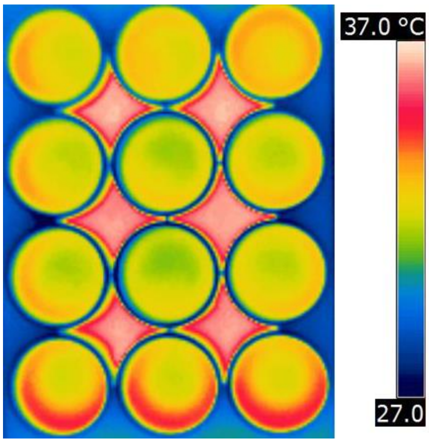

Supplementary Figure 12. Methodology for infrared thermography of brown adipocytes in culture. Example of thermographic recording of brown adipocytes in culture as described in Methods.

Western blot figure 1 b:

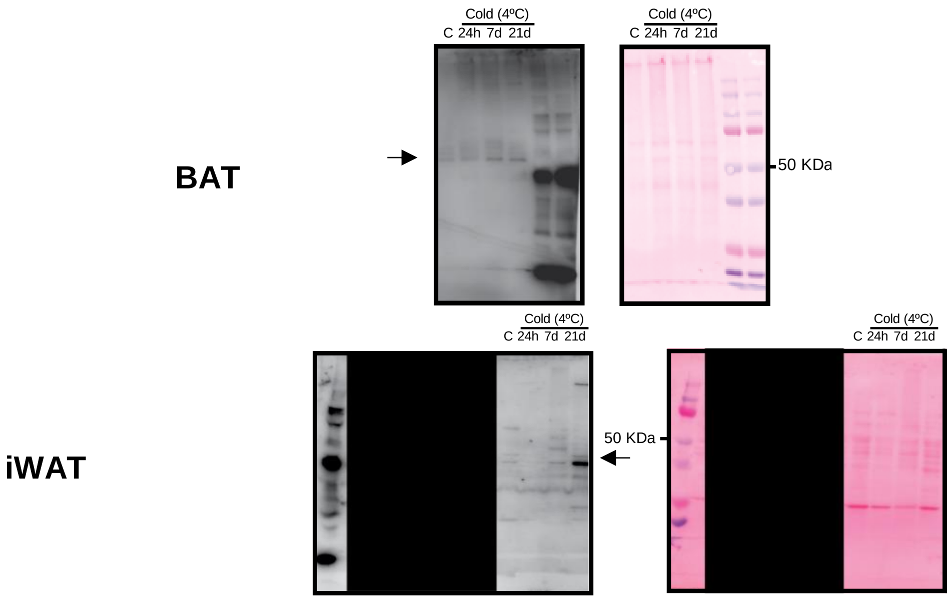

Western blot figure 1 f:

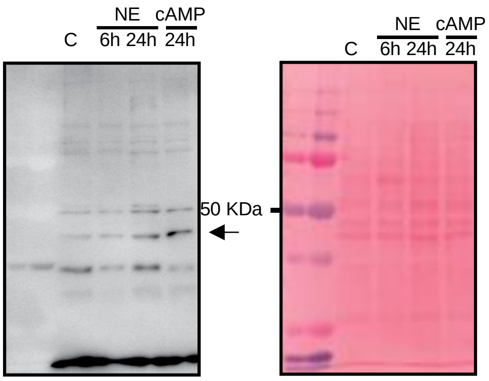

Western blot figure 2:

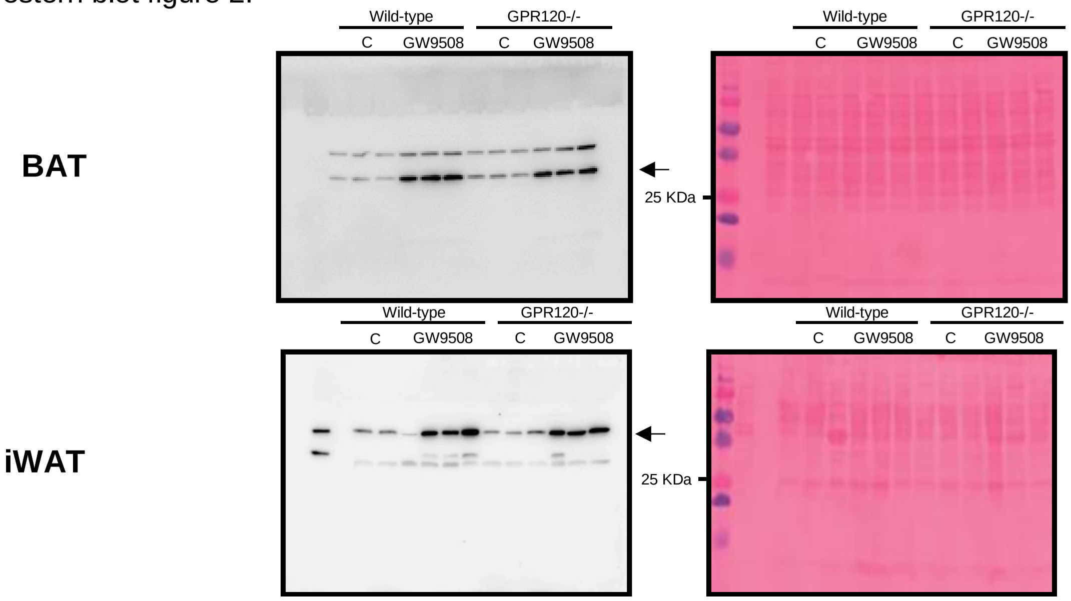

Supplementary Figure 13. Uncropped western blots and markers. Immunoblots (left) and Ponceau-stained membranes showing weight markers (right). Arrow, specific immunoblot signal. Black areas, lanes unrelated to current study.

Supplementary Table 1. Tissue weight, body temperature and circulating metabolic and hormonal parameters in mice treated with dietary GW9508 for 7 days Adult mice were fed a standard chow diet supplemented with or without GW9508 for 7 days. Data are means ± s.e.m. (*P<0.05 relative to untreated control mice; two-tailed unpaired student t-test).

	Control (n=5)	GW9508(n=6)
**Body Weight (g)**	29.0 ± 0.8	27,6 ± 0.6
**Food intake (g)**	6.4 ± 0.7	4.8 ± 0.6
**iBAT (mg)**	99.4 ± 7.8	96.0 ± 3.1
**iWAT (mg)**	95.4 ± 14	100.2 ± 11.8
**eWAT (mg)**	180.0 ± 24.7	181.1 ± 25.9
**mWAT (mg)**	169.1 ± 27.5	133.4 ± 13.0
**Rectal temperature (ºC)**	35.7 ± 0.4	35.8 ± 0.4
**Core temperature (eye) (ºC)**	35.9 ± 0.5	35.6 ± 0.3
**Glucose (mg/dL)**	183 ± 10	157 ± 14
**Triglycerides (mg/dL)**	140 ± 14	169 ± 12
**Insulin (pg/dL)**	966 ± 99	633 ± 62 *
**IL-6 (pg/dL)**	54 ± 39	27 ± 11
**Leptin (pg/dL)**	1090 ± 382	1374 ± 324
**PAI1 (pg/dL)**	1168 ± 334	1579 ± 329
**Resistin (pg/dL)**	1930 ± 130	1971 ± 171

Supplementary Table 2. Tissue weight, body temperature and circulating metabolic and hormonal parameters in wild-type mice and GPR120-null mice treated with dietary GW9508 for 7 days Adult mice were fed a standard chow diet supplemented with or without GW9508 for 7 days. Data are means ± s.e.m. (*P<0.05 relative to untreated control mice; ANOVA with Tukey's post hoc test).

	**Wild-type**		**GPR120-/-**	
	**Control**	**GW9508**	**Control**	**GW9508**
**Body weight (g)**	27.7 ± 0.6	27.0 ± 0.6	26.9 ± 0.5	27.4 ± 0.3
**Food intake (g)**	4.0 ± 0.2	4.5 ± 0.1	4.3 ± 0.1	4.1 ± 0.21
**iBAT (mg)**	83.7 ± 1.8	97.4 ± 15.5	90.6 ± 5.4	91.6 ± 7.4
**iWAT (mg)**	147.6 ± 10.0	205.5 ± 3.0	133.4 ± 7.3	204.0 ± 31.9
**eWAT(mg)**	264.6 ± 15.3	440.5 ± 143.5	228.8 ± 21.5	433.8 ± 50.0
**Core temperature (eye) (ºC)**	35.8 ± 0,5	35.6 ± 0,3	36.2 ± 0,18	36.0 ± 0.2
**Glucose (mg/dL)**	195 ± 11	185 ± 8	170 ± 9	174 ± 17
**Triglycerides (mg/dL)**	159 ± 13	138 ± 7	157 ± 9	160 ± 19
**Insulin (pg/dL)**	1520 ± 45	996 ± 96 *	1110 ± 350	1200 ± 180

Supplementary Table 3. TaqMan probes (Applied Biosystems) used for RT-PCR quantification of gene transcripts.

Gene	Reference
18S rRNA	Hs99999901_s1
FGF21	Mm00840165_g1
PGC-1alpha	Mm447183_m1
UCP1	Mm00494069_m1
Glut1	Mm00441480_m1
PRDM16	Mm00712556_m1
Bmp8b	Mm00432115_g1
CoxIV	Mm00438289_g1
Sirt3	Mm00452129_m1
PPARg	Mm440945_m1
FABP4	Mm00445880_m1
GPR120	Mm00725193_m1
GPR40	Mm00809442_s2
Leptin	Mm00434759_m1
PPIA(cyclophillin)	Mm02342430_g1
